# A review of psychological research on *kando* as an inclusive concept of moving experiences

**DOI:** 10.3389/fpsyg.2022.974220

**Published:** 2022-10-17

**Authors:** Shoko Yasuda, Haruka Shoda, Ai Uemiya, Tadao Isaka

**Affiliations:** ^1^Mori Arinori Institute for Higher Education and Global Mobility, Hitotsubashi University, Kunitachi, Japan; ^2^Faculty of Sport and Health Science, Ritsumeikan University, Kusatsu, Japan; ^3^Faculty of Human Sciences, Institute of Human and Social Sciences, Kanazawa University, Kanazawa, Japan

**Keywords:** *kando*, being moved, awe, *kama muta*, generative mechanism, emotions, Japan

## Abstract

We are emotionally moved when we give birth to a child, witness the triumph of an athlete, listen to a beautiful piece of music, and so forth. Such moving experiences have been described as a form of emotion, by terms such as being moved, awe, and *kama muta*, each of which have been studied as a separate, but interrelated, psychological phenomenon. Japanese people use the term *kando* to describe these experiences collectively. In this study, we propose that *kando* should be treated as an umbrella term covering being moved, awe, and *kama muta*. To this end, we reviewed the literature on *kando* conducted by Japanese researchers and compared it to relevant concepts, mainly examined in Western countries. We also reviewed the literature on the generative mechanism of *kando*, and established that emotional and physical reactions are important to determine the degree of *kando* across the cases with and without storyline. Furthermore, individual characteristics such as trait empathy may generate a stronger degree of *kando*. *Kando* experiences can affect subsequent behaviors and cognition, though we still need evidence that such change can be triggered genuinely by the experience of *kando*. We suggest that *kando* may make our neuro-cognitive network tend toward more direct, unconscious, and impulsive decision making. One of the remaining questions in this domain is whether the mental construct of *kando* can find an equivalent to people in Western countries. For this purpose, events and reactions relevant to *kando* experiences should be systematically collected from a broad population.

## 1. Introduction

When parents hold their newborn baby in their arms, they feel delightful pleasure which transcends worries about the future. We may experience tears of joy when we are occasionally touched by the kindness of others. We may get emotionally moved when we see athletes overcome adversity and win a match. These kinds of complex and basically positive experiences have been reported cross-culturally in the domain of interpersonal relationships, and the concept of “*kama muta*” (i.e., “being moved by love” in Sanskrit) has been proposed in recent years as a framework to describe such social emotion (e.g., Fiske et al., 2019). In this paper, we would like to introduce *kando* (
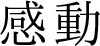
 in Japanese letters) as a hierarchically broader framework describing such experiences. A part of the research on *kando* overlaps with that on human relationships, such as experiencing human kindness (i.e., *kama muta*, Zickfeld et al., 2020), or observing altruistic moral behaviors (i.e., elevation, Silvers and Haidt, 2008). We also express *kando* when we perceive the vastness of a spectacular view (i.e., awe, Nakayama et al., 2020) or when an unknown piece of music seeps into our hearts and lingers in the mind for a long time (Yasuda and Nakamura, 2008; Yasuda et al., 2020). Adding to this stream of research, we propose in this paper that *kando* can be a construct encompassing multiple mental activities, some of which are relevant to interpersonal relationships while others can be triggered by events or objects with weak (or perhaps no) connection to such social mechanisms.

The term *kando* is used regularly in Japan, and also in Chinese (
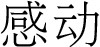
, gǎndòng) and Korean (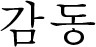, gamdong) languages with different pronunciations. The meaning of *kando* as per a Japanese dictionary (Shinmura, 2018) is “to feel something deeply and be moved by it.” Considering studies that are mainly conducted in Western countries (e.g., Zickfeld et al., 2019), the concepts of “being moved,” awe, and *kama muta* seem analogous to that of *kando*, but the nuances are not completely matched.

In this review, we introduce the phenomenon of what native Japanese speakers (and perhaps people in China and Korea) call *kando* by comparing it with relevant Western concepts. Since *kando* is a Japanese word, much of the literature on it has been written in Japanese. However, the concept of *kando* could be an important universal phenomenon, and therefore, we would like to highlight the significance of writing this review in English. We first discuss commonalities and differences between *kando* and other concepts of *kando*-related experiences (We henceforth refer to “being moved,” “awe,” and “*kama muta*” collectively, as “*kando*-related experiences”). Second, we introduce studies mainly conducted with the Japanese population, in which researchers explored the mechanism of *kando* for cases with storyline (e.g., novel, movie) and without storyline (e.g., music). Third, we consider individual characteristics (e.g., trait empathy) that are likely to cause *kando*. Fourth, we explore possible functions of *kando* in human behavior and cognitive processes. Finally, we propose some questions for future research.

## 2. Comparing *kando* and its relevant concepts

People in Japan experience *kando* in their everyday life. When people experience or see other people experiencing *kando*, consistent patterns of psychological, behavioral, and physiological reactions occur. Existing literature has mentioned that “being moved,” “awe,” and “*kama muta*” share the meaning of *kando*, with commonalities in a person's psychological reactions (Kato and Murata, 2017; Maeura et al., 2020). Several researchers in Japan have explored experiences of *kando* through both theoretical and empirical approaches, but have still been unsuccessful in defining it (Maeura et al., 2020). To provide a valid and comprehensive definition of *kando*, we will need further data collected in a bottom-up manner to determine situations, events, and reactions in *kando* experiences, which is outside the scope of the present study. In other words, the aim of this review is not to provide the precise definition of *kando*, but to describe the collective knowledge on *kando* as an Asian concept that has been examined by researchers in Japan for the last three decades. By doing so, we would position *kando* in the research stream of *kando*-related concepts, which have been examined mainly by Western researchers. This review can also provide a foundation for further discussion in terms of the universalities and culture-specific aspects of *kando* experiences, from which we should in time, be able to provide the precise definition of *kando* through future research.

A series of studies by Tokaji (e.g., Tokaji, 2001, 2003) can be considered representative of *kando* research in Japan^1^. Tokaji (2001) pointed out that reactions of *kando* are strong and last for a relatively long time. He noted that the characteristics of *kando* are not in concordance with any type of human affect (e.g., emotion, mood, sentiment). *Kando* is separate from emotion, but emotions are associated with the generation of *kando* (Tokaji, 2001). He also mentioned that though positive emotions are experienced when feeling *kando*, negative emotions can co-occur in the generative process. It indicates that *kando* is a complex and mixed mental representation and cannot be simply modeled on the conventional framework of emotion.

*Kando* is the broad concept behind the moving experiences of people, so that, plausibly, it is a construct of multiple mental activities. *Kando* is triggered by a wide variety of events and objects from one's everyday life (e.g., it stopped raining just before leaving home, the traffic signals on the way were all green) to a special event occurring a few times within one's lifetime (e.g., marriage, the birth of a child, winning a great competition) (Oode et al., 2007; Maeura et al., 2020). In this meaning, *kando* experiences can be brought about by deviations from individuals' expectations, which are formed by the accumulation of experiences, stored in long-term memory. For example, *kando* can range from sudden surprises or instances of luck that rarely occur in everyday life (e.g., one's feeling as if some enigmatic but great power moved them) to one's greatest happiness or pleasure that is finally achieved after long and often painful processes (Oode et al., 2007). The psychological phenomenon of *kando* can cover a spectrum of triggering events and intensities of the reactions to them.

The concepts equivalent to *kando* outside Japan (at least in Western countries) have not been examined empirically. One reason for this is that an English equivalent, or at least, a noun “expression of the language” cannot be found (Tokaji, 2001). When people express a concept like *kando* in English, they usually use passive verbs such as “being moved,” “being impressed,” and “being touched” (Tokaji, 2001). However, there has been no agreement on whether these English expressions correspond to it. In Zickfeld et al. (2019), the degree of *kama muta* when watching videos was rated by people from various nations. For Japanese participants, the researchers translated “being moved” (one item in the *kama muta* scale) into *kando*, and the *kama muta* score by the Japanese participants was the least among the 11 nations (see Table 8 in Zickfeld et al., 2019). The result of this experiment may suggest that the meaning of “being moved” is not equivalent to that of *kando*. To determine whether *kando* can be interchangeably used as “being moved,” we should examine the equivalence of events and reactions related to *kando* and *kando*-related experiences in Japan and other countries, respectively.

Various kinds of *kando*-related experiences have been studied in theoretical and empirical research and the events described in literature that trigger such experiences vary enormously. Menninghaus et al. (2015) showed in a German population that “being moved” can be evoked by a variety of events, including interpersonal relationships, critical life events, political and social events, nature, arts, and others. Cova and Deonna (2014) argue that “being moved” occurs in situations where positive core values are prominent. Positive core values refer to that which is fundamentally important to humans, such as brotherhood, solidarity, peace, health, virtuosity, and beauty (Cullhed, 2020). As in Menninghaus et al. (2015), experiences of “being moved” can be triggered by events with levels of low to moderate arousal, but the reactions are very strong. On the contrary, the reactions of *kando* ranged from relatively weak to extremely strong degrees (Oode et al., 2007), regardless of the event's levels of arousal. In other words, *kando* includes psychological states with relatively weak reactions, while “being moved” refers to a stronger psychological state. This can be one of the points differentiating between *kando* and “being moved.”

In recent years, commonalities and differences between awe and *kando* have also been examined. Awe indicates, for example, a state when a person is exposed to a great work of art, intellectual inspiration, or the beauty of nature (Shiota et al., 2007). A pioneering study of awe is Keltner and Haidt (2003), in which they identified two central components: vastness and accommodation. Vastness refers not only to size in a physical sense, but also in a social one, including fame, authority, or prestige. It refers to a person's perception of a larger existence when compared to themselves and their internal standards. Accommodation refers to the need to adapt a person's cognitive framework post confronting a larger entity. Previous literature has pointed out that awe is a complex psychological state (Chirico et al., 2016) in that awe cannot be categorized into the dimensional framework of emotion (pleasant–unpleasant emotion dimension) (Arcangeli et al., 2020). Awe is often regarded as a positive emotion in that it generally has the ability to improve mental well-being as well as enhance prosocial behavior, life satisfaction, and meaning in life (e.g., Shiota et al., 2011; Campos et al., 2013). Nevertheless, awe often occurs along with negative emotions, such as fear of a larger entity or recognition of one's own smallness, indicating that awe is a construct of complex emotional experiences, and cannot be simply categorized into the frameworks of basic emotions (Arcangeli et al., 2020; Nakayama et al., 2020). In short, awe is a complex psychological state that differs from emotions expressed by single adjectives, which is akin to the case of *kando*. On the other hand, the intensity of awe is powerful (Chirico et al., 2016), and its concept involves strong emotional experiences with a sense of “magnitude of existence” or “beyond comprehension” (Maeura et al., 2020). In this view, *kando* and awe are different, in that, according to Maeura et al. (2020), the intensity of *kando* ranges from mild to strong.

As another concept relevant to *kando, kama muta* has been studied intensively in recent years. *Kama muta* means “moved by love” in Sanskrit, and is a technical term to explain moving experiences incorporating the relational models theory (Fiske et al., 2019). In the series of studies, the researchers decided to avoid using the folk expressions of “being moved,” “being touched,” or moving experiences, and this is the reason of why the term *kama muta* is proposed, by which they have begun the scientific approach. They have modeled *kama muta* as a core, hierarchically higher, concept behind emotions like “being moved,” “being touched,” and so forth, and this theorized emotion can be implemented by different cultures in different ways (Fiske et al., 2019). Theoretically, *kama muta* occurs when communal sharing relationships (hereafter “CSR”) are suddenly intensified (Fiske et al., 2019). For example, *kama muta* is likely to be experienced by witnessing the kind behaviors of others. Communal sharing relationships refers to the process by which people are able to love, unite, and integrate. In other words, the trigger for the *kama muta* is mostly prosocial and altruistic (Zickfeld et al., 2019). Furthermore, according to Fiske et al. (2019), *kama muta* can explain many culturally evolved practices, institutions, roles, narratives, arts, and artifacts by regarding experiences of the great nature (Petersen et al., 2019) or music (even instrumental, non-lyric music, Vuoskoski et al., 2022) as ones containing social connectedness, or quasi-social relationships. We acknowledge that these kinds of explanations are theoretically possible as a mechanism, but we still should explore whether possible events or objects irrelevant to CSR lead to *kando* and *kando*-related experiences. For example, we experience *kando* when we are captivated accidentally by the beauty of a single tone in walking down a road, when we get goosebumps just by being exposed to the first notes of music, when we are overwhelmed by an abstract painting, or when we win the lottery. It seems unreasonable to assume that *kando* and *kando*-related experiences in all these experiences are originated by CSR.

The theory of *kama muta* defines it as a core concept behind related emotions such as nostalgia, longing, patriotism, warming of the heart (see Figure 1 in Fiske et al., 2019), whereas we consider *kando* to be generated as an outcome by multiple mental processes, as we describe in section 3. This can be a major difference between *kando* and *kama muta*, even though we acknowledge that the concept of *kama muta* overlaps with that of *kando*. According to Zickfeld et al. (2020), *kama muta* often co-occurs with different emotions, including negative ones (e.g., sadness, anxiety), but *kama muta* itself is considered a positive experience, which is in line with the aforementioned characteristic of *kando* described by Tokaji (2020).

We need further research to elucidate whether the mental representation of *kando* exists in people from different countries. Further theoretical research is also needed to understand the differences between *kando* and relevant concepts such as “being moved,” awe, and *kama muta*. By doing so, we will understand the universality of *kando*, as a term describing the whole experience ranging from weak to strong intensities, but still special for individuals.

## 3. Generative mechanism of *kando*: Cases of story and music

*Kando* is triggered by a wide variety of events and objects. As Tokaji (2003) mentioned, triggers of *kando* can be broadly classified into cases with a storyline (e.g., movies, TV dramas) and without one (e.g., natural scenery, works of art). Tokaji (2003) modeled the process of generating *kando* evoked by events with a storyline. According to this model, *kando* arises when we experience tension psychologically (or anxiety) and physically at the moment when the story unfolds, which then will be released by further development of the story. In other words, for cases with a storyline, negative emotions are needed in the process of generating *kando*. In this process, individual characteristics (e.g., trait empathy) affect the transitions in emotion and physical reactions preceding the generation of *kando*.

Yasuda et al. (2020) investigated empirically how *kando* is generated by listening to music (i.e., cases without storyline). They showed a statistical model in which listeners' perception of acoustic features (i.e., loudness, pitch, tempo) determines their physical reactions and emotions, and then, such reactions induce the listener's *kando*. Also, the listener's characteristic of trait empathy involves the generative process of physical reactions, emotions, and *kando*. The statistical model indicates significantly that *kando* is a “consequence” of physical reactions and emotions, with a better fit than models in which physical reactions and emotions occur as a result of *kando* (Yasuda, 2010). This model supports our assumption that *kando* is generated as an outcome of some mental processes, apart from the theory of *kama muta* which supposes various emotions to be derivative forms of a core concept.

The two models of *kando* have been provided by distinct studies (Tokaji, 2003; Yasuda et al., 2020), but reveal commonalities across the cases with and without a storyline. First, physical reactions and emotions occur prior to the generation of *kando*. Second, trait empathy and other individual characteristics influence the process of generating *kando*. In the following sections, we focus on these two points and introduce literature on the relationship among *kando*, physical reactions, and emotions, as well as on individual characteristics relevant to *kando*.

### 3.1. Physical reactions and emotions in *kando*

The relationship between *kando* and physical reactions has been examined, with many reports concerning *kando* through listening to music. Nishizono (1981) qualitatively examined the state of *kando* caused by music, indicating that it is accompanied by physical reactions such as changes in heart rate and respiration. In Ohgushi (2000), music-major undergraduates described their experiences of *kando* by listening to music, and they frequently reported physical reactions such as goosebumps, racing heart, tears, and shivers down the spine. Furthermore, Yasuda and Nakamura (2008) quantitatively showed that five physical reactions (i.e., goosebumps, lump in the throat, shivers down the spine, being close to tears, excitement) were experienced frequently during *kando* when listening to music.

In several studies conducted in Western countries, associations between *kando*-related experiences and physical reactions are also shown with similarities to those in *kando*. In Landmann et al. (2019), chills, tears, and warmth in the chest occurred more frequently when watching movies that moved the audience than those that did not. The autonomic nervous system, as measured by increases in heart rate, respiration rate, respiration depth, and continuous phasic skin conductance, becomes more activated when the participants feel *kama muta* experiences (Zickfeld et al., 2020). Zickfeld et al. (2020) conducted an experiment using videos targeting *kama muta* and measured participants' physiological reactions throughout watching the videos. They compared measures during segments eliciting weak *kama muta* to those eliciting strong ones. The results indicated that stronger experiences of *kama muta* induce decreased heart rate, deepening breathing, a higher number of relative non-specific skin conductance, stronger zygomaticus activity, higher skin temperature, and goosebumps. The results suggest that sympathetic and parasympathetic nervous systems were activated alternately while viewing the videos. As in the aforementioned models (Tokaji, 2003), *kando* is evoked through the transition from mental and physical tension to its release. The co-occurrence of sympathetic and parasympathetic nervous activities observed in the study of *kama muta* (Zickfeld et al., 2020) also fits well with the generative model of *kando* by Tokaji (2003).

There has been much debate as to whether *kando* is a type of emotion. Emotions are generally described through the theory of basic emotions or dimensional structure (Barrett and Russell, 2015). The basic emotion theory classifies emotions into several major categories such as anger, fear, sadness, enjoyment, disgust, and surprise (e.g., Ekman, 1992, 1999). However, *kando* is often not accompanied by an emotion with a single adjective but is related to multiple emotion words. Therefore, *kando* is a complex psychological representation that cannot be defined in a framework of basic emotions (Tokaji, 2020). It is also difficult to explain *kando* in the framework of the dimensional model of emotion (Russell, 1980), in which emotion consists of valence and arousal dimensions. Though *kando* is a positive experience, it can be accompanied by negative emotions in the generative process (Kato and Murata, 2013). For example, we often experience *kando* with achievements, which comes after overcoming negative emotions as associated with hardships and difficulties. Possibly, these negative experiences can be transformed over time into *kando* accompanied by positive emotions. Accordingly, it is implausible to place *kando* at a single point in the two-dimensional emotion space. In the generative process of *kando*, several types of emotions can co-occur, and *kando* itself cannot be explained by the existing models of emotion.

### 3.2. Individual differences in reactions to *kando*

Existing literature have reported that individual characteristics (e.g., trait empathy) are involved in the generation of *kando*. Hashimoto and Ogura (2002) examined the relationship between *kando* and trait empathy based on adolescents' free descriptions, and showed that among the components of trait empathy, “understanding the position of others” and “altruistic concerns” are strongly related to *kando*. People with these particular components of trait empathy, have dispositions that allow them to take a positive interest in others and the world around them, and to try to understand their mind. Also, in Zickfeld et al. (2017), a meta-analysis of 16 studies was conducted in which feelings of either “being moved” or “being touched” was induced through films, stories, or episodes, and the association with the trait empathy was examined. The results showed that the score of “feeling moved,” which is a composition of ratings for “being moved and touched,” is strongly associated with the participant's “empathic concern” [one of four factors of the Interpersonal Reactivity Index (IRI), Davis, 1983], which is other-oriented feelings of sympathy and concerns about those who are less fortunate (Davis, 1983). *Kando* includes the psychological state of *kama muta* in which one is moved by prosocial or altruistic behaviors. Given this assumption, it seems natural that the interpersonal aspect of trait empathy would be associated with *kando*.

Films, stories, and episodes that elicit *kando* usually have a clear narrative with a storyline. In contrast, music often evokes *kando*, even though it does not explicitly contain a storyline. According to Eerola et al. (2016), listeners who are moved more by listening to unfamiliar sad music are likely to have a “fantasy” personality (i.e., one of the factors of the IRI, indicating a tendency to transpose themselves imaginatively into the feelings and actions of fictitious characters in books, movies, and plays). Even when the storyline is not clearly described in the musical work, people who have abundant imagination can compensate for it by developing a story, which evokes stronger *kando* in listeners. In other words, people with stronger sense of “fantasy” or similar trait of empathy may tend to imagine a fictional story with social relationships in their mind by listening to music, by which they may experience a stronger CSR. This might be the reason of why *kama muta* is generated by listening to music, as shown by Vuoskoski et al. (2022).

## 4. *Kando* and its functions

Next, we consider possible functions of *kando* in human social behaviors and the cognitive processes. According to Tokaji (2004), the functions of *kando* are categorized into the following three groups: (1) other-oriented thinking and interpersonal acceptance, (2) motivation, and (3) update of one's cognitive framework. Researchers in Western countries have mainly investigated the social functions (corresponding to other-oriented thinking and interpersonal acceptance in Tokaji, 2004) through the studies of elevation. Elevation is defined as the emotion experienced when witnessing virtuous acts of remarkable moral goodness (Haidt, 2003; Pohling and Diessner, 2016; Thomson and Siegel, 2017). According to Fiske et al. (2019), the difference between elevation and *kama muta* is mainly in the nomenclature, even though their detailed definitions differ (i.e., elevation is as for a third-person behavior, but *kama muta* includes both one's own experience as well as observation of a third-person experience). Since *kando* is a concept that includes *kama muta*, elevation can also be regarded as one of the components of *kando*. Elevation can trigger altruistic and moral behaviors (Schnall et al., 2010) as well as strengthen relationships with others (e.g. Fiske et al., 2019). For example, Freeman et al. (2009) showed that by reading a story of white people helping black people, participant donation behaviors toward Black-oriented charity tended to increase. However, it is too simplistic to conclude that emotionally moving experiences cause prosocial, altruistic, and moral motivations and behaviors. Some researchers suggest that current results might be confounded by other factors (Landmann, 2021). *Kando* is not only other-oriented, but sometimes, it can be a more intuitive and self-absorbed phenomenon that leads one to the opposite side of deep reflection.

As compared with the studies presented above, fewer studies have examined the functions of *kando* in light of cognitive processes. Slater et al. (2019) examined that the association between emotionally moving experiences and the tendency to await a reward in a delayed discounting task. The participants were presented with eudaimonic and non-eudaimonic narrative films, and rated their degrees of “poignancy,” “being emotionally moved” (i.e., touched, moved), and “moral elevation” (i.e., inspired, meaningful). According to the results, the degrees of “being emotionally moved” after watching eudaimonic narrative films negatively correlated with the tendency to wait for the reward, whereas participants with higher ratings of “poignancy” (i.e., negative emotion) showed a tendency to prefer the delayed reward. This indicated that an emotionally moving experience by itself may induce an impulsive cognitive process.

We can assume that there are two types of functions of *kando*—a short-term and a long-term effect. First, the short-term effect of *kando* can be caused by impulsive cognitive process. As aforementioned, *kando* is a psychological phenomenon accompanied by emotions, which can be the main factors causing the short-term effects generated in the *kando* experience. The processes of emotion and cognition are deeply associated with each other and the influence of emotion on cognition has been discussed in various models (e.g., Bower, 1981; Damásio, 1994). Among them, in an evolutionary model of emotional functioning, emotions exert their effects in situations where immediate decision-making is required (e.g., Damásio, 1994; Cosmides and Tooby, 2000). In addition, several studies have suggested that positive emotions are associated with intuitive, heuristic cognitive processing, while negative emotions promote analytic, controlling processing (e.g., Forgas, 2001). In this light, immediately after the *kando* experience, people might be led to an intuitive cognitive process such as the findings observed in Slater et al. (2019).

The aforementioned research that showed altruistic and moral behaviors after emotionally moving experiences (e.g., Freeman et al., 2009; Schnall et al., 2010) may also be relevant to the intuitive cognitive process. Landmann (2021) suggested that when “being moved” by a specific situation, the participant's behavior and the motive behind it seem to resemble the context or situation that they were moved by, and this effect may not be generalized to other domains. For example, the experience of “being moved” by moral virtues may increase helping behaviors toward others. If we are moved by seeing someone achieving success in the Olympic games, we might be motivated to a behavior directed to self-improvement. In other words, moving experiences tend to induce more biased behaviors and thoughts, which are determined by the presented context or situation. As evidence, the previous studies have used a procedure in which the participants responded regarding their motive or their behavior immediately after the emotionally moving experience (e.g., Freeman et al., 2009; Schnall et al., 2010). This “intuitive” decision-making may be evoked by the emotion involved in the process of *kando* experience. Landmann (2021) also pointed out the “dark side” of emotionally moving experiences in that such experiences have effects of manipulating and inducing behavior similar to the preceding stimuli. If the short-term effects of *kando* are automatic, intuitive, and sometimes impulsive, the experience could hasten decision making. For example, an experience of *kando* through an unknown wonderful technology may make people purchase an expensive product, which they would normally hesitate to purchase if they went through an analytical thought process. *Kando* experienced by the courageous actions of others may also give one the courage to do something that one would not usually do.

Nevertheless, Tokaji (2004) showed that the effects of *kando* are self-reflective, and the accumulation of *kando* experiences promotes the individual's growth and changes one's cognitive framework. This can be the long-term effects of *kando*, which can be obtained through a deliberate cognitive process and can be explained in line with autobiographical memory research. When people experience *kando*, they share the experience with others, or recall it repeatedly to themselves from time to time. In this process, they try to think about the meaning of the event (or create memories about the *kando* experiences). We try to interpret and make sense of the *kando* experience by which we evaluate it, relate it to our identity and life story, and/or reconstruct the memory. This process is called “autobiographical reasoning” (McLean and Fournier, 2008), which is an introspective cognitive process of recalling the past and interpreting and evaluating the events (memories). As stated previously, it also involves the reconstruction of memory in a form consistent with one's current identity. The experience of *kando* through an analytical thinking process, such as autobiographical reasoning, transforms cognitive frameworks through transforming the interpretation of events (or memory).

Studies of autobiographical memory showed that memories vividly recalled with strong emotions are more likely to involve autobiographical reasoning. Memories that involve much autobiographical reasoning eventually becomes important in the person's life, and are called self-defining memories (McLean and Thorne, 2003). Self-defining memories are sometimes described as the memory of events that are turning points in one's life. The studies on *kando* and emotionally moving experiences apply approaches commonly used in the study of autobiographical memory, such as asking participants to recall their own *kando* experiences (e.g., Oode et al., 2007; Menninghaus et al., 2015). Further research is needed to elucidate the whole picture regarding the cognitive process of *kando*, involving human decision making, working memory, executive function, and episodic memory (e.g., autobiographical memory).

## 5. Discussion

We have reviewed the literature on *kando*, which is an inclusive but partially distinct construct of “being moved,” awe, and *kama muta*, concepts which have been explored mainly in Western countries (e.g., Chirico et al., 2016; Fiske et al., 2019; Zickfeld et al., 2019, 2020). Whether these concepts are concordant with *kando* is still unknown. In this paper, we discussed that *kando* should be treated as an umbrella concept including “being moved,” awe, and *kama muta*. In general, researchers in this domain have explored generative mechanisms of *kando* and *kando*-related experiences, assuming that these experiences are in common across different cultures (i.e., “etic” aspects in linguistics). In future work, empirical research should be carried out to explore roles of culture in these experiences (i.e., “emic” aspects). We, as Japanese, can accept, understand, and share such a broad concept as *kando*. This may be due to cultural pragmatics. As Nisbett et al. (2001) described, compared to thought styles of Westerners, those of East Asians (including Japanese) are likely to be holistic, such as attending to the entire environment (rather than the main objects), making relatively little use of formal logic and categorization, and relying on dialectical reasoning. From another viewpoint, Japan can be categorized as a “high-context” culture, in which statements are less explicit in communication, and people are more likely to read the messages embedded in the context (Hall, 1976; Kitayama and Ishii, 2002). In terms of these culturally determined manners, Japanese people are likely to keep things ambiguous, obscure, and inconclusive, due to their holistic thinking and high-context communication styles. This might explain why a single term, *kando*, is easy to use in Japan, without expressing each detail of the experiences and emotions with multiple adjectives.

In the literature on *kama muta* (Fiske et al., 2019), the researchers avoid using the folk concept of “moving” as a technical term, and a scientifically explorable *kama muta* has been treated as the core concept explaining the human experience with communal sharing. We acknowledge that introducing a novel concept requires a clear scope and range of what it deals with (i.e., *kando*) and constructs the basis for exploring the mechanism of the phenomenon. However, as we described so far, *kando* experiences vary as a function of contexts, targets, and individual characteristics (e.g., trait empathy), so that *kando* cannot be represented with a single mental model. A single framework is likely to be insufficient to cover a range of *kando* experiences, but plausibly, *kando* should be represented as multiple categories in the mind. Thus, as our next step of research, we should clarify and definitize the categories of what we call *kando*, and by doing so, scientific research will be made possible by clarifying the target categories (e.g., *kando* with music, *kando* with achievements). By accumulating such attempts, our understanding of universalities and domain-specific features of *kando* will expand greatly.

To understand the categories of *kando*, the necessary and sufficient conditions for recognizing it should be elucidated. First, we should confirm situations in which *kando* is triggered in a broader and more systematic manner. In Japan, and perhaps in other Asian countries sharing this expression (i.e., China, Korea), we experience *kando* from minor daily occurrences (e.g., listening to lovely music, “the traffic lights were all green!”) to an event occurring only occasionally in a lifetime (e.g., the birth of a child). The existing research portfolio is still insufficient to depict the whole picture of episodes causing *kando* as well as the human characteristics relevant to *kando*. To this end, it is effective to collect descriptions of events in which people have experienced *kando*, ranging from the everyday to major life accomplishments.

Furthermore, there can be great individual differences in the experience of *kando*, in that an event that causes a *kando* response in one individual may not be perceived so by another individual. One of the future research questions should be whether we can assume the existence of a common script in everyone's mind that causes *kando*, or whether such a script does not exist and *kando* can be completely a personal experience. In this viewpoint, we should identify variables that can explain individual differences in the experience of *kando*.

As another direction for study, we should confirm externally observable parameters to measure a state of *kando* (e.g., behavior, physiological responses, neuro-cognitive activity) or, at least, subjective psychological indicators. We assume that *kando* is different from general human emotions as described in the basic emotion theory or the dimensional theory of emotion. *Kando* is a multifaceted reaction described with multiple adjectives. In line with the literature on *kama muta* (Fiske et al., 2019; Zickfeld et al., 2019), some experiences of *kando* can occur with positive emotions following experiences of negative, painful, or grief emotions, suggesting that the mechanism of *kando* cannot be explained merely by the basic emotion theory. Also, *kando* cannot be expressed on a continuum of a few emotional dimensions, such as valence and arousal (Tokaji, 2003), because *kando* is often expressed with mixed emotion words, sometimes with contrasting meanings such as “bittersweet.” It is desirable to approach the psychological experience unique to *kando* in terms of both theoretical and empirical perspectives.

Finally, it would be interesting to explore the evolutionary and adaptive significance of *kando* in humans. For example, one's *kando* experience at the birth of a child or grandchild can be a function to drive the parents or grandparents to care for the newborn babies. When adolescents struggle to be independent of their parents, they become absorbed in music, animation, and videos (North and Hargreaves, 1999), by which they may gain strong *kando* experiences. Such effects of dissipating negative experiences peculiar to adolescence will affect their psychological development or formation of identity throughout their lifetime. Moreover, the accumulation of daily experiences of *kando*—by watching a baseball game at a stadium, watching a movie at a theater, or attending a live music concert—functions as mood regulation. People who are constantly pursuing technological advances (e.g., smartphones, 8K televisions) aim to satisfy themselves by updating and accumulating their *kando* experiences, indicating that the continuous pursuit of *kando* experiences can be a major factor in people's decision making. In general, the reward system network works when humans experience such pleasant and deep feelings like *kando*, so that the reactions after experiencing *kando* become relevant to the reward system. Both the cognitive process and the outcomes of *kando* should be explored in future research.

## Author contributions

SY wrote the first draft of the manuscript. HS and AU wrote sections of the manuscript. TI supervised the authors and contributed to the conception of the work. All authors contributed to manuscript revision, read, and approved the submitted version.

## Funding

The authors declare that this study received funding from Yamaha Motor Co., Ltd. The funder was not involved in the study design, collection, analysis, interpretation of data, the writing of this article, or the decision to submit it for publication.

## Conflict of interest

This work was a part of collaborative research (*kando* project) with Yamaha Motor Co., Ltd. However the company was not involved in the writing of this article and the decision to submit it for publication. The authors declare that the research was conducted in the absence of any commercial or financial relationships that could be construed as a potential conflict of interest.

## Publisher's note

All claims expressed in this article are solely those of the authors and do not necessarily represent those of their affiliated organizations, or those of the publisher, the editors and the reviewers. Any product that may be evaluated in this article, or claim that may be made by its manufacturer, is not guaranteed or endorsed by the publisher.

## Footnotes

^1^Tokaji (2003) referred to *kando* as *kandoh*. These two are different notations for the same word ().
